# Asylums in India

**Published:** 1894-07-21

**Authors:** 


### ASYLUMS IN INDIA.

### VI.—BURMA : THE RANGOON ASYLUM.

The lunatic asylum at Rangoon appears to have suffered from the depletion of the Provincial Medical Department resulting from the military operations in Burma, and although the proposal that a resident assistant surgeon should be appointed has received the sanction of Government, the services of an officer of the class required have not yet been available. On the criminal side this institution is still overcrowded, and an increasingly large number of criminal insane had to be confined in the Rangoon Jail. A new asylum is to be opened shortly at Mandalay, and the accommodation will then be ample for the province.

On January 1st, 1891, there were 251 patients under treatment, and 84 were admitted during the year, giving a total population of 335 insane; of these 32 were discharged cured, six improved, and 21 "otherwise"—which in this case includes four escapes—and 22 died, so that 254 remained under treatment at the close of the year. The daily average number was 248·62, and the percentage of cures to this average was 12·87, as compared with 10·79 in 1890, and 13·9 for the previous ten years. The death-rate stood at 8·05, which is a marked improvement on 1890, when it stood at 15·41; the average death-rate for the previous ten years was 10·38.

The total expenditure of the year was Rs.28,341 15 7; the following statement shows all the most important features of this brief but adequate report:—

| | | | | |
|---|---|---|---|---|
| 1. Average daily number of inmates... ... | 248·62 | | | |
| 2. Average daily number employed ... ... | 219 | | | |
| 3. Total expenditure throughout the year ... | Rs.28,341 | 15 | 7 | |
| 4. Total income from lunatic labour, &c. ... | 5,809 | 10 | 5 | |
| 5. Payment by friends of lunatics ... ... | 249 | 0 | 0 | |
| 6. Received from municipalities ... ... | 11,222 | 4 | 4 | |
| 7. Average cost per patient, as per column 3 | 114 | 0 | 0 | |
| 8. Cost per patient, after deducting 5 and 6... | 67 | 13 | 9 | |

With the exception of lamp-lighting, all work connected with the asylum builings and grounds is done by the patients. They are further employed in the asylum on coir-pounding, basket making, broom making, and carpentry, and out of doors in planting and cutting hedges, weeding, cleaning compounds, cutting grass, and making private roads. The number employed is very satisfactory.

If a resident assistant surgeon is appointed, and some material improvements, such as raising the height of the outer wall so as to prevent all possibility of escape, are effected, this asylum will have a very satisfactory account to give of itself. Even under the present disadvantages this report testifies to good work.

